# Comparative Evaluation of Cold Dissection (Suture vs. Ligation) and Hot Dissection Tonsillectomy in Children: Postoperative Pain, Bleeding, and Operative Time

**DOI:** 10.3390/jcm14186491

**Published:** 2025-09-15

**Authors:** Ismail Aytac, Berkay Güzel, Orhan Tunc, Elif Baysal, Fatih Ubeydullah Bescocuklu

**Affiliations:** 1 Department of Otorhinolaryngology, School of Medicine, Gaziantep University, 27310 Gaziantep, Turkey; orhantip@hotmail.com (O.T.); baysalelif@yahoo.com (E.B.); fubeydullah@gmail.com (F.U.B.); 2 Department of Otorhinolaryngology, Basaksehir Cam and Sakura City Hospital, 34480 Istanbul, Turkey; drberkayguzel@gmail.com

**Keywords:** tonsillectomy, cold dissection, ligation, cautery, postoperative pain, hemorrhage, pediatrics

## Abstract

**Objectives***:* Tonsillectomy is among the most frequently performed pediatric ENT procedures. Post-tonsillectomy pain and hemorrhage remain key determinants of postoperative morbidity and may differ by surgical technique. This work’s objective is to compare postoperative pain, bleeding, and operative duration across three pediatric tonsillectomy techniques: cold dissection with suturing, cold dissection with ligation, and hot dissection with bipolar cautery. **Materials and Methods***:* In this single-center, prospective study, 150 children (*n* = 50 per group) undergoing tonsillectomy (with adenoidectomy) between October 2022 and October 2024 were assigned preoperatively to the following groups: Group 1—cold dissection + suturing; Group 2—cold dissection + ligation; Group 3—hot dissection (bipolar cautery). Pain was assessed with the Wong–Baker FACES scale at 1, 6, and 24 h, days 3 and 7, and with the Parents’ Postoperative Pain Measure (PPPM) at 1, 6, and 24 h. Primary bleeding was defined within 24 h; secondary bleeding was within 2 weeks. Operative time was recorded from first incision to hemostasis. Non-parametric tests and chi-square analyses were used with *p* < 0.05 considered significant. **Results***:* Of 150 patients, 58% were male. No primary hemorrhage occurred. Secondary hemorrhage occurred in 4/150 (2.7%): 1/50 (2%) in Group 1, 0/50 (0%) in Group 2, and 3/50 (6%) in Group 3 (overall *p* > 0.05). Readmission for oral-intake difficulty occurred in 4/150 (2.7%): 1/50 (2%) in Group 1 and 3/50 (6%) in Group 3 (*p* > 0.05). Operative time differed significantly across groups (Kruskal–Wallis *p* < 0.05), being longest in Group 1 and shortest in Group 3 (17.53 ± 1.26 min); Group 2 averaged 18.60 ± 0.94 min and Group 1 21.89 ± 1.64 min. Pain decreased over time in all groups (Friedman *p* < 0.001). Across virtually all time points, Group 2 (ligation) had significantly lower Wong–Baker and PPPM scores than Groups 1 and 3 (Dunn post-hoc, adjusted *p* < 0.05), while Groups 1 and 3 did not differ consistently. **Conclusions**: Cold dissection with ligation yielded the most favorable pain profile while maintaining low bleeding rates; hot dissection minimized operative time but tended toward higher secondary bleeding and postoperative intake difficulties. Technique selection should prioritize postoperative comfort and morbidity reduction—particularly in pediatric populations—favoring cold dissection, with ligation offering a consistent analgesic advantage.

## 1. Introduction

Tonsillectomy is one of the most commonly performed surgical procedures in otorhinolaryngology, particularly in pediatric populations, and remains a cornerstone in the management of recurrent tonsillitis and obstructive sleep-disordered breathing [1]. Post-tonsillectomy bleeding (PTB) is among the most significant complications, as it may result in emergency intervention, rehospitalization, and—in rare cases—life-threatening outcomes. Therefore, clear classification of PTB into primary (within 24 h) and secondary (after 24 h) hemorrhage, as well as identification of risk factors, is essential for optimizing patient care [2].

Multiple surgical techniques have been developed and refined with the aim of reducing intraoperative and postoperative complications, minimizing short-term morbidity, and decreasing operative times [3]. These techniques can be broadly divided into cold dissection methods (such as steel instruments with suture or ligation for hemostasis) and hot dissection methods (including monopolar or bipolar electrocautery, coblation, and radiofrequency devices) [4,5,6,7]. While cold dissection has been the historical standard, the optimal technique remains controversial, with no universally accepted “gold standard” established to date.

Postoperative hemorrhage incidence varies considerably in the literature, ranging from 1% to 20%. A recent systematic review and meta-analysis by Alenezi et al. confirmed this variability, highlighting significant heterogeneity among different surgical techniques and patient populations [4,5,6,7,8]. Some studies have reported increased secondary bleeding risk with hot techniques, leading to recommendations in favor of cold dissection [9,10]. Conversely, other investigations have found no significant difference in bleeding rates between hot and cold techniques [11]. A number of comparative studies have evaluated cold dissection against bipolar diathermy or coblation, with mixed results [11,12,13].

Proponents of hot dissection techniques highlight advantages such as improved intraoperative hemostasis, reduced operative blood loss, and shorter surgery times [14]. However, histopathological evidence suggests that cold dissection causes less thermal damage to tonsillar tissue, potentially reducing postoperative pain and inflammatory sequelae. For example, a retrospective cohort study conducted at the University of Oviedo in 2016, including 240 pediatric patients, found that cold dissection was associated with lower complication rates and fewer emergency department visits compared to electrocautery [15]. Similarly, Xu et al. reported a 14% secondary bleeding rate for bipolar diathermy versus no bleeding in the cold dissection group [16].

A further limitation of the existing literature is that most studies have compared only two techniques at a time. To our knowledge, few randomized trials have directly compared three commonly used approaches in a single study, which is a distinguishing feature of the present work. Given the ongoing debate and the lack of consensus, further comparative studies are warranted. The present study aims to evaluate postoperative pain, frequency of postoperative bleeding, and operative time among three tonsillectomy approaches—cold dissection with suturing, cold dissection with ligation, and hot dissection using bipolar cautery—in pediatric patients undergoing adenotonsillectomy.

## 2. Materials and Methods

### 2.1. Study Design and Study Population

The study was approved by the Institutional Ethics Committee (decision no: 2022/195, date: 28 September 2022). This prospective, single-center clinical study was conducted in the Otorhinolaryngology Department of a tertiary care hospital between October 2022 and October 2024. The study protocol was approved by the institutional ethics committee, and written informed consent was obtained from the parents or legal guardians of all participants prior to enrollment.

A total of 150 pediatric patients scheduled for adenotonsillectomy were included. Inclusion criteria were as follows: (1) age 3–15 years; (2) indication for tonsillectomy due to recurrent tonsillitis or obstructive sleep-disordered breathing; and (3) suitability for general anesthesia. Indication type (infection vs. sleep-disordered breathing) was recorded for each patient; however, subgroup analyses were not powered due to limited sample sizes. Exclusion criteria were as follows: (1) acute upper respiratory tract infection at the time of surgery; (2) known coagulopathy; (3) tonsillar neoplasm; (4) craniofacial anomalies; and (5) chronic medical comorbidities potentially influencing perioperative outcomes (e.g., asthma, obesity, diabetes mellitus).

### 2.2. Randomization and Groups

Eligible patients were randomized preoperatively into three equal groups (*n* = 50 each) using a computer-generated random number table, with allocation concealed in sealed opaque envelopes. Stratification by age or sex was not performed; however, subsequent analysis confirmed comparable baseline demographic characteristics across groups:

➢ Group 1 (Cold Dissection + Suturing): Standard cold-knife dissection was performed, followed by hemostasis using absorbable suture material (Vicryl 3-0, Ethicon) to approximate the anterior and posterior tonsillar pillars. No local anesthetic or topical hemostatic agents were applied.
➢ Group 2 (Cold Dissection + Ligation): Standard cold dissection was performed, and bleeding vessels were ligated with the same absorbable suture material (Vicryl 3-0, Ethicon).
➢ Group 3 (Hot Dissection—Bipolar Cautery): Bipolar cautery dissection was used for tissue separation and hemostasis, with energy settings adjusted to minimize thermal injury.

All procedures were performed under standardized general anesthesia by surgeons with comparable experience. In all groups, oral intake was initiated at the 4th postoperative hour. Patients were discharged on postoperative day 1 with prescriptions for oral amoxicillin–clavulanate and paracetamol.

### 2.3. Outcome Measures

**Figure 1:**
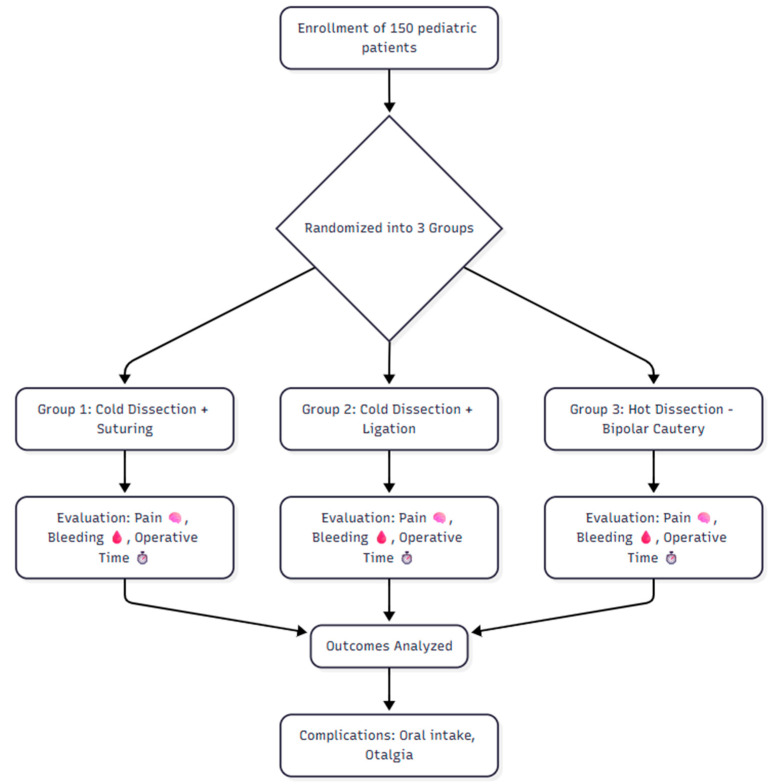
Flowchart of study.

1. Postoperative Pain:
  ➢ Wong–Baker FACES Pain Rating Scale was used at postoperative hours 1, 6, and 24, and on days 3 and 7.
  ➢ Parents’ Postoperative Pain Measure (PPPM) was administered at hours 1, 6, and 24 before meals, fluid intake, or analgesic use.
2. Postoperative Bleeding:
  ➢ Primary bleeding: Any hemorrhage within 24 h post-surgery.
  ➢ Secondary bleeding: Any hemorrhage occurring from 24 h to 14 days postoperatively. All bleeding events were initially assessed by the operating surgeon during hospitalization and subsequently confirmed through outpatient follow-up visits or standardized parent reports, with final adjudication by an independent ENT consultant.
3. Secondary Outcomes
  ➢ Operative Time: Measured in minutes from the first incision to the completion of hemostasis.
  ➢ Complications: Any postoperative events including readmission for oral intake difficulty, infection, or other adverse events (Figure 1).

### 2.4. Statistical Analysis

A priori sample size calculation was performed based on expected differences in Wong–Baker pain scores between groups. Assuming a medium effect size (f = 0.25), α = 0.05, and power (1 − β) = 0.80, a minimum of 42 patients per group was required. To compensate for potential dropouts, we included 50 patients per group (total *n* = 150). All statistical analyses were performed using IBM SPSS Statistics for Windows, Version 27.0 (IBM Corp., Armonk, NY, USA). Continuous variables were first assessed for normality using the Shapiro–Wilk test. Normally distributed data were expressed as mean ± standard deviation (SD), whereas non-normally distributed data were expressed as median (minimum–maximum). Categorical variables were summarized as counts and percentages. Between-group comparisons of continuous variables were conducted using the Kruskal–Wallis H test for independent samples, with Dunn’s test and Bonferroni-adjusted *p*-values for post hoc pairwise comparisons. Within-group changes over repeated measurements were analyzed using the Friedman test, followed by Dunn–Bonferroni post hoc analysis when appropriate. Categorical variables were compared using the Chi-square test or Fisher’s exact test (when ≥20% of expected frequencies were <5). Effect sizes (η^2^ for non-parametric tests, Cramer’s V for categorical variables) and 95% confidence intervals (CI) were calculated where relevant to aid interpretation of clinical significance. A two-tailed *p*-value < 0.05 was considered statistically significant for all analyses.

## 3. Results

A total of 150 pediatric patients were included in the study, with 50 patients in each group. The proportion of male patients was 62.0% in Group 1 (*n* = 31), 58.0% in Group 2 (*n* = 29), and 54.0% in Group 3 (*n* = 27), with no statistically significant difference between groups (*p* = 0.771). Primary bleeding did not occur in any patient across all groups. Secondary bleeding was observed in one patient (2.0%) in Group 1, none in Group 2, and three patients (6.0%) in Group 3, and there was no statistically significant difference among the groups (*p* = 0.167). Postoperative oral intake difficulty occurred in one patient (2.0%) in Group 1, none in Group 2, and three patients (6.0%) in Group 3, without a significant difference (*p* = 0.167). Postoperative otalgia was reported in one patient (2.0%) in Group 1, none in Group 2, and two patients (4.0%) in Group 3, also showing no statistically significant difference (*p* = 0.364) (Table 1).

The mean operative time was longest in Group 1 (cold dissection with suturing) at 21.89 ± 1.64 min (median: 22.10 min), followed by Group 2 (cold dissection with ligation) at 18.60 ± 0.94 min (median: 18.30 min), and shortest in Group 3 (hot dissection with bipolar cautery) at 17.53 ± 1.26 min (median: 17.20 min). The difference in operative times among the three groups was statistically significant (*p* = 0.001) (Table 2).

Postoperative pain scores, assessed using the Wong–Baker FACES Pain Rating Scale, showed statistically significant differences among the three groups at all evaluated time points (Hour 1, Hour 6, Hour 24, Day 3, and Day 7; all *p* < 0.005). At Hour 1, the median (IQR) pain scores were 4.0 (3.0–5.0) in Group 1, 3.0 (3.0–4.0) in Group 2, and 4.0 (4.0–5.0) in Group 3. Group 2 consistently exhibited lower pain scores across all time points compared to Groups 1 and 3, while Group 3 generally had the highest scores, particularly in the early postoperative period. Similarly, Parents’ Postoperative Pain Measure (PPPM) scores differed significantly among the groups at hour 1, hour 6, and hour 24 (all *p* < 0.005). Median (IQR) PPPM scores at hour 1 were 10.0 (8.0–11.0) for Group 1, 7.0 (6.0–9.0) for Group 2, and 11.0 (9.0–12.0) for Group 3. Group 2 consistently demonstrated lower PPPM scores at each measured time point (Table 3).

Pairwise comparisons using the Dunn–Bonferroni method revealed that Group 2 (cold dissection with ligation) had significantly lower pain scores compared to Group 1 (cold dissection with suturing) at Hour 1 (*p* = 0.001) and Day 3 (*p* = 0.010). Group 2 also reported significantly lower scores than Group 3 (hot dissection with bipolar cautery) at Hour 1 (*p* < 0.001), Hour 6 (*p* = 0.002), Hour 24 (*p* = 0.003), Day 3 (*p* < 0.001), and Day 7 (*p* = 0.002). There were no statistically significant differences between Group 1 and Group 3 at any time point (all adjusted *p* > 0.05) (Table 4).

Dunn–Bonferroni pairwise comparisons demonstrated that Group 2 (cold dissection with ligation) had significantly lower PPPM scores compared to Group 1 (cold dissection with suturing) at Hour 1 (*p* < 0.001), Hour 6 (*p* = 0.010), and Hour 24 (*p* = 0.039). Group 2 also showed markedly lower scores than Group 3 (hot dissection with bipolar cautery) at all evaluated time points: Hour 1 (*p* < 0.001), Hour 6 (*p* < 0.001), and Hour 24 (*p* = 0.003). There were no statistically significant differences between Group 1 and Group 3 at any time point (all adjusted *p* > 0.05) (Table 5).

## 4. Discussion

In this prospective, randomized comparison of three tonsillectomy techniques—cold dissection with suturing, cold dissection with ligation, and hot dissection using bipolar cautery—we found that cold dissection with ligation was associated with significantly lower postoperative pain scores at almost all evaluated time points, without increasing the incidence of postoperative bleeding. Conversely, hot dissection provided the shortest operative time but was associated with a secondary hemorrhage rate of 6% (3/50), compared with 2% (1/50) in the suturing group and 0% (0/50) in the ligation group. Although these differences did not reach statistical significance, they suggest a potential tendency toward higher bleeding risk with hot dissection, alongside increased postoperative oral intake difficulties. However, these results must be interpreted cautiously, as our sample size was not sufficient to make definitive conclusions about bleeding risk. Our observed rates fall within the ranges reported in large pediatric series for cold tonsillectomy (2–6%) and monopolar cautery (≈3–4%), while some high-volume centers performing thousands of procedures annually have reported rates as low as 1–2%. Thus, robust evaluation of bleeding risk requires larger multi-center cohorts or institutional registries with high case volumes.

Post-tonsillectomy hemorrhage (PTH) is one of the most critical complications in pediatric ENT surgery, potentially leading to readmission, reoperation, and rare life-threatening outcomes [1,5]. Our secondary bleeding rate (2.7%) was in the lower range reported in large pediatric series, such as Attya and Ünsal et al. documented rates between 1% and 6% [4,17].

Several studies have indicated higher secondary bleeding rates with hot dissection methods compared to cold dissection [8,10,16]. Similarly, we observed more secondary hemorrhage in the hot dissection group, although the difference was not statistically significant—likely due to the limited number of events.

Importantly, our ligation group had zero cases of secondary bleeding, consistent with Bashir and Swami [18], who demonstrated superior hemostatic security with silk ligation compared to bipolar cautery. Mechanical vessel closure avoids thermal injury to surrounding tissues and thereby reduces the risk of delayed bleeding. This finding also emphasizes the importance of a meticulous hemostatic technique in minimizing post-tonsillectomy hemorrhage [11,19].

Pain control is essential for recovery, oral intake, and quality of life after tonsillectomy, especially in children [13,20]. Our study found significantly lower Wong–Baker and PPPM scores in the ligation group compared to both suturing and hot dissection across most time points. Mathúna et al. concluded in a meta-analysis that knot-tying hemostasis is associated with reduced postoperative discomfort compared to cautery [21]. It should also be acknowledged that postoperative analgesic protocols may influence reported pain outcomes; although standardized regimens were applied across all groups, variations in parental adherence and individual analgesic response could have contributed to differences in perceived pain. Furthermore, the indication for tonsillectomy (recurrent infection versus sleep-disordered breathing) is another factor known to affect pain perception, with prior studies showing less pain after surgery for infection than for sleep apnea. In our cohort, both indications were included and distributed evenly across groups, but the analysis was not stratified by indication due to sample size limitations.

Hot dissection techniques, although efficient for hemostasis, can cause greater thermal injury, leading to prolonged inflammation and nociceptor activation [14,22]. Bhankhodia et al. and Rubinstein & Derkay reported higher pain scores with electrocautery and other thermal devices [23,24]. Alenezi et al. further supported that cold dissection techniques limit tissue trauma, explaining their more favorable pain profiles [8]. The slightly higher pain scores in our suturing group compared to ligation may be due to increased tissue manipulation during suture placement.

As expected, hot dissection significantly reduced operative time compared to both cold dissection techniques, in line with findings from Iqbal et al. and Kandemir et al. [22,25]. Although shorter operative duration may be advantageous in minimizing anesthesia exposure—particularly in high-risk pediatric populations [1,15]—this potential benefit should be carefully weighed against the increased likelihood of postoperative bleeding and discomfort associated with thermal techniques [24,26].

Although the primary focus of our study was on postoperative bleeding and pain, it is important to acknowledge that tonsillectomy can be associated with a wide spectrum of other complications. These include respiratory events [1], rare but severe conditions such as subcutaneous emphysema [7], and anesthesia-related morbidity [12]. Evidence from large-scale analyses, including those by Anwaegbu et al., indicates that institutional protocols, patient selection, and surgeon experience are critical determinants of postoperative complication rates [27].

Our findings suggest that cold dissection with ligation offers the most favorable balance between safety, pain control, and operative efficiency in pediatric tonsillectomy. Hot dissection may be advantageous in settings where operative time is critical, but clinicians should remain aware of its potential trade-offs. Lao et al. and Rubinstein & Derkay have emphasized that surgical technique choice should be individualized based on patient-specific factors, institutional resources, and surgeon expertise [24,28].

Unlike most prior studies comparing only two tonsillectomy techniques, our randomized trial simultaneously evaluated three approaches—cold dissection with suturing, cold dissection with ligation, and hot dissection using bipolar cautery—under standardized surgical and perioperative conditions. This three-arm design represents a key novelty of the study, and its contribution should be considered in light of ongoing debates regarding the optimal tonsillectomy method. Previous research often emphasized operative time or bleeding [22,25] without detailed, repeated pain assessments. We incorporated both Wong–Baker and PPPM scales, providing a more comprehensive analysis of postoperative discomfort. While Bashir and Swami and Xu et al. reported lower bleeding with ligation, our study confirmed zero secondary hemorrhage in this group, strengthening evidence for its safety in pediatric patients [16,18]. By evaluating pain, bleeding, and operative time together in a single cohort, our study offers a broader comparative perspective to guide surgical technique selection.

### Limitations of the Study

This study has some limitations. First, being conducted in a single tertiary center may limit the generalizability of the findings to other populations and practices. Second, although the sample size was sufficient for detecting differences in pain scores and operative times, the low incidence of postoperative hemorrhage reduced the power to assess bleeding rates between techniques. Third, despite comparable surgeon experience, subtle differences in surgical skill or intraoperative decisions could have influenced outcomes. Fourth, follow-up was limited to the early postoperative period, and late complications beyond two weeks were not assessed. Fifth, patient- and parent-reported pain scores, although validated, remain subjective and may be influenced by age and psychological factors. Furthermore, blinding was not feasible, which may have introduced unconscious parental bias and implicit evaluator bias. The wide age range (3–15 years) is another limitation, as pain perception differs between young children and adolescents; age-specific analyses were not possible. In addition, no serial observation of suture integrity was performed in the suturing group, and potential breakdown may have increased discomfort during oral intake. Finally, the sample size (*n* = 50 per group) was too small to draw firm conclusions about bleeding risk. Although our rates align with those reported in the literature, larger multicenter studies or high-volume institutional registries are needed for robust comparisons.

## 5. Conclusions

In conclusion, cold dissection with ligation was associated with the most favorable postoperative pain profile and no secondary hemorrhage, while maintaining acceptable operative times in pediatric tonsillectomy. Hot dissection using bipolar cautery provided the shortest operative duration but showed a tendency toward increased postoperative discomfort and higher rates of secondary bleeding. Cold dissection with suturing demonstrated intermediate outcomes for both pain and operative time. These findings suggest that surgical technique selection should be individualized, balancing the need for operative efficiency with the priority of minimizing postoperative morbidity—particularly in younger or high-risk pediatric populations. Further multi-center studies with larger sample sizes are warranted to validate these results and to explore the long-term outcomes of each technique. Our findings support prioritizing cold dissection with ligation as a safe and analgesically superior option for pediatric tonsillectomy, pending confirmation in multi-center trials.

## Figures and Tables

**Table 1:** Demographic characteristics and early postoperative outcomes of pediatric patients undergoing tonsillectomy with different surgical techniques.

Variables	Group 1	Group 2	Group 3	*p* Value
	N (%)			
Gender				0.771 ᵃ
Male	31 (62.0)	29 (58.0)	27 (54.0)	
Female	19 (38.0)	21 (42.0)	23 (46.0)	
Primary bleeding				—
No	50 (100)	50 (100)	50 (100)	
Secondary bleeding				0.167 ᵃ
No	49 (98.0)	50 (100)	47 (94.0)	
Yes	1 (2.0)	0 (0)	3 (6.0)	
Postoperative oral intake difficulty				0.167 ᵃ
No	49 (98.0)	50 (100)	47 (94.0)	
Yes	1 (2.0)	0 (0)	3 (6.0)	
Postoperative otalgia				0.364 ᵃ
No	49 (98.0)	50 (100)	48 (96.0)	
Yes	1 (2.0)	0 (0)	2 (4.0)	

ᵃ Chi-square test. Data are presented as *n* (%). Group 1: cold dissection with suturing, Group 2: cold dissection with ligation, 3. Group 3: hot dissection with bipolar cautery.

**Table 2:** Operative time comparison among pediatric tonsillectomy techniques.

Group	Surgical Technique	Mean ± SD (min)	Median (min)	*p* Value ᵃ
1	Cold dissection + suturing	21.89 ± 1.64	22.10	0.001 *
2	Cold dissection + ligation	18.60 ± 0.94	18.30	
3	Hot dissection (bipolar cautery)	17.53 ± 1.26	17.20	

ᵃ Kruskal–Wallis test. SD: standard deviation. * Statistically significant at *p* < 0.05

**Table 3:** Postoperative pain scores by time point according to surgical technique (Wong–Baker FACES Pain Rating Scale and Parents’ Postoperative Pain Measure—PPPM).

Time Point	Group 1	Group 2	Group 3	*p* Value ᵃ^,^*
	**Wong–Baker FACES Pain Rating Scale** **Median (IQR)**			
Hour 1	4.0 (3.0–5.0)	3.0 (3.0–4.0)	4.0 (4.0–5.0)	0.001 *, η^2^ = 0.12
Hour 6	3.0 (2.0–4.0)	3.0 (2.0–3.0)	3.0 (3.0–4.0)	0.002 *
Hour 24	2.0 (2.0–3.0)	2.0 (1.0–2.0)	3.0 (2.0–3.0)	0.004 *
Day 3	2.0 (1.0–2.0)	1.0 (1.0–2.0)	2.0 (1.0–3.0)	0.001 *
Day 7	1.0 (0–1.0)	0.0 (0–1.0)	1.0 (1.0–2.0)	0.003 *
	**PPPM Median (IQR)**			
Hour 1	10.0 (8.0–11.0)	7.0 (6.0–9.0)	11.0 (9.0–12.0)	0.001 *
Hour 6	9.0 (7.0–10.0)	6.0 (5.0–8.0)	9.0 (8.0–10.0)	0.001 *
Hour 24	8.0 (6.0–9.0)	6.0 (5.0–7.0)	8.0 (7.0–9.0)	0.003 *

* *p* < 0.05; Effect sizes are presented as η^2^ for non-parametric tests and Cramer’s V for categorical variables. ᵃ Kruskal–Wallis test. IQR: interquartile range.

**Table 4:** Post-hoc pairwise comparisons of Wong–Baker FACES Pain Rating Scale scores between surgical techniques at each time point.

Time Point	Comparison	Median Difference (95% CI)	Adjusted z	Adj. *p* Value ᵃ
Hour 1	Group 2 vs. Group 1	1.0 (0.5–1.5)	3.54	0.001 *, η^2^ = 0.12
	Group 2 vs. Group 3	−1.0 (−1.5–−0.5)	−4.66	0.000 *, η^2^ = 0.18
	Group 1 vs. Group 3	−0.5 (−1.0–0.5)	−1.15	0.750, η^2^ = 0.02
Hour 6	Group 2 vs. Group 1	0.0 (−0.5–0.5)	1.38	0.507
	Group 2 vs. Group 3	−1.0 (−1.5–−0.5)	−3.48	0.002 *
	Group 1 vs. Group 3	−0.5 (−1.0–0.0)	−2.13	0.099
Hour 24	Group 2 vs. Group 1	−0.5 (−1.0–0.0)	2.00	0.136
	Group 2 vs. Group 3	−1.0 (−1.5–−0.5)	−3.29	0.003 *
	Group 1 vs. Group 3	−0.5 (−1.0–0.5)	−1.31	0.571
Day 3	Group 2 vs. Group 1	−1.0 (−1.5–−0.5)	2.94	0.010 *
	Group 2 vs. Group 3	−1.0 (−1.5–−0.5)	−3.87	0.000 *
	Group 1 vs. Group 3	−0.5 (−1.0–0.5)	−0.96	1.000
Day 7	Group 2 vs. Group 1	0.0 (−0.5–0.5)	1.35	0.533
	Group 2 vs. Group 3	−1.0 (−1.5–−0.5)	−3.39	0.002 *
	Group 1 vs. Group 3	−0.5 (−1.0–0.5)	−2.07	0.117

ᵃ Dunn–Bonferroni adjusted *p* values. * *p* < 0.05; Effect sizes are presented as η^2^ (non-parametric) alongside pairwise comparisons.

**Table 5:** Post-hoc pairwise comparisons of Parents’ Postoperative Pain Measure (PPPM) scores between surgical techniques at each time point.

Time Point	Comparison	Median Difference (95% CI)	Adjusted z	Adj. *p* Value ᵃ
Hour 1	Group 2 vs. Group 1	2.0 (1.0–3.0)	3.81	0.000 *, η^2^ = 0.15
	Group 2 vs. Group 3	−3.0 (−4.0–−2.0)	−4.72	0.000 *, η^2^ = 0.20
	Group 1 vs. Group 3	−1.0 (−2.0–0.0)	−0.94	1.000 *, η^2^ = 0.04
Hour 6	Group 2 vs. Group 1	2.0 (1.0–3.0)	2.93	0.010 *
	Group 2 vs. Group 3	−3.0 (−4.0–−2.0)	−4.04	0.000 *
	Group 1 vs. Group 3	−1.0 (−2.0–0.0)	−1.13	0.780
Hour 24	Group 2 vs. Group 1	−2.0 (−3.0–−1.0)	−2.49	0.039 *
	Group 2 vs. Group 3	2.0 (1.0–3.0)	3.25	0.003 *
	Group 1 vs. Group 3	0.0 (−1.0–1.0)	0.76	1.000

ᵃ Dunn–Bonferroni adjusted *p* values. * *p* < 0.05; Effect sizes are presented as η^2^ for non-parametric comparisons.

## Data Availability

De-identified data are available from the corresponding author upon reasonable request. Public sharing is restricted to protect pediatric patient privacy and to comply with institutional ethics approval.
